# Effect of Prolonged Radiotherapy Treatment Time on Survival Outcomes after Intensity-Modulated Radiation Therapy in Nasopharyngeal Carcinoma

**DOI:** 10.1371/journal.pone.0141332

**Published:** 2015-10-27

**Authors:** Pei-Jing Li, Ting Jin, Dong-Hua Luo, Ting Shen, Dong-Mei Mai, Wei-Han Hu, Hao-Yuan Mo

**Affiliations:** 1 Department of Nasopharyngeal Carcinoma, Sun Yat-sen University Cancer Center, State Key Laboratory of Oncology in South China, Collaborative Innovation Center for Cancer Medicine, Guangzhou, People’s Republic of China; 2 Department of Radiation Oncology, Zhejiang Cancer Hospital, Hangzhou, Zhejiang, People’s Republic of China; 3 Zhejiang Key Laboratory of Radiation Oncology, Hangzhou, Zhejiang, People’s Republic of China; 4 Department of Oncology and Hematology, The people’s Hospital of Nanhai District, Foshan, People’s Republic of China; Duke Cancer Institute, UNITED STATES

## Abstract

**Purpose:**

To estimate the influence of prolonged radiation treatment time (RTT) on survival outcomes in nasopharyngeal carcinoma after continuous intensity-modulated radiation therapy.

**Methods and Materials:**

Retrospectively review 321 patients with NPC treated between October 2009 and December 2010 and all of them underwent simultaneous accelerated intensity-modulated radiation therapy. The fractionated dose was 2–2.47 Gy/F (median 2.27 Gy), and the total dose for nasopharyngeal region was 64–74 Gy/ 28–33 fractions. The association of prolonged RTT and treatment interruption with PFS, LRFS and DFFS were assessed by univariate analysis and multivariate analysis. Survival analyses were carried out using Kaplan–Meier methodology and the log-rank test was used to assess the difference. The Cox regression proportional hazard model was used for multivariate analyses and evaluating the prognostic parameters for PFS, LRFS and DFFS.

**Results:**

Univariate analysis revealed no significant associations between prolonged RTT and PFS, LRFS, DFFS when dichotomized using various cut-off values (all *P*>0.05). In multivariate analysis, RTT (range, 36–63 days) as a continuous variable, had no influence on any survival outcome as well (*P*>0.05). T and N classification were independent prognostic factors for PFS, LRFS and DFFS (all *P*<0.05, except T classification for LRFS, *P* = 0.057). Age was an independent prognostic factor for PFS (hazard ratio [HR], 1.033; *P* = 0.008) and DFFS (HR, 1.032; *P* = 0.043).

**Conclusion:**

We conclude that no such association between survival outcomes and radiation treatment duration (range: 36–63 days) can be found in the present retrospective study, however, we have to remind that prolongation in treatment should be limited in clinical application and interruptions caused by any reason should be minimized as much as possible.

## Introduction

Nasopharyngeal carcinoma (NPC) has an extremely uneven endemic distribution within Southern China and Southeast Asia [1]. The last two decades have witnessed key milestones in the treatment of NPC and continual improvements in treatment outcomes. As it is radiosensitive and in an anatomically-complex location, radiotherapy remains the main treatment modality for NPC [2]. Significant improvements in therapeutic effect were achieved with the extensive application of intensity-modulated radiotherapy (IMRT) and addition of concurrent chemotherapy to radiotherapy; advancements in imaging technology have also led to improved outcomes [3–5]. The 3-year local control rate for NPC after IMRT is approximately 84% to 95% and the 3-year overall survival rate ranges from 85% to 90% [6–9]. Overall survival varies considerably depending on tumor stage; on aggregate, approximately 76%-80% of patients survive at least 5years [5, 10, 11].

Guidelines from the U.S. National Comprehensive Cancer Network recommend concurrent chemoradiotherapy (CCRT) in the presence or absence of adjuvant chemotherapy as the first-line treatment for NPCs. Although the benefit of adjuvant chemotherapy is still open to debate, adjuvant chemotherapy is commonly prescribed for patients with locally advanced NPC at our institution and is well tolerated [12, 13]. However adjuvant chemotherapy may increase the risk of treatment interruptions.

Interruptions are inevitable during treatment, due to treatment-related toxicity, holidays, machinery faults and other causes. The effect of the total irradiation time on treatment outcomes has recently been emphasized in other cancers [14–17]. An extension of treatment time has been reported with an undesirable effect on local control and/or overall survival in cervical carcinoma, prostate carcinoma, non-small cell carcinoma of the lung and carcinoma of the larynx. Multiple retrospective studies and randomized clinical trials have demonstrated that a protracted treatment time would contribute to inferior local control and overall survival in head and neck cancer (HNC) patients with radiotherapy alone [18–25]. Accelerated repopulation by tumor cells is the assumed radiobiological explanation for the poor prognosis of a prolonged treatment time, especially in patients with rapidly-proliferating tumor types such as HNCs. In head and neck cancers, tumor clonogen repopulation occurs as a burst that—on average—starts around the third to fifth week after the initiation of radiotherapy. The stimulation of radiotherapy decreases the tumor clonogen doubling time from approximately 60 days to 4 days by the middle of treatment [26].

NPC is a distinct type of head and neck cancer, there still have been conflicting results regarding the effect of a prolonged radiation therapy time in NPC. Adverse effects of prolonged treatment time on NPC patients treated with two-dimensional radiation (2DRT) had been reported [27–31]; however, it has rarely been investigated whether it is necessary to strictly control the radiotherapy time for patients with NPC in the IMRT era. Additionally, much more work needs to be done such as to establish a suitable criteria as reference when a patient in discomfort needs interruption in radiotherapy and to find methods to communicate with patients about such interruptions. We conducted this retrospective study to evaluate the relationship between the radiation treatment time (RTT) and therapeutic effects in patients treated using IMRT and provide practical recommendations for the management of radiation treatment interruptions in NPC.

## Methods and Materials

### Patient characteristics and treatment

The inclusion criteria for this study were as follows:

1. *Histology*: histologically-proven undifferentiated or non-keratinizing squamous cell carcinoma of the nasopharynx.
2. *Stage*: I-IVB NPC. All patients were restaged using the 7th edition of the American Joint Committee on Cancer (AJCC) staging system. Tumor staging was based on routine examinations (physical examination, nasopharyngeal fiberoptic endoscopy, chest X-ray, abdominal sonography, magnetic resonance imaging [MRI], bone scan, positron emission tomography-computed tomography [PET-CT]).
3. *Treatment modality*: Patients treated using radical IMRT with or without platinum-based concurrent chemotherapy; patients who received either induction chemotherapy or adjuvant chemotherapy were excluded.
4. *Radiation*: The gross tumor volume (GTV) was contoured according to the tumor presentation on images and endoscopic findings. The clinical tumor volume, CTV1, included the whole nasopharynx and GTV with a 5–10 mm margin (including at least all of the nasopharyngeal mucous layer and tissues within 5 mm). The CTV2 covered the CTV1 with a 5–10 mm margin for high-risk local structures, positive lymph nodes, the GTVnd and areas of lymphatic drainage. Patients who were treated using an anterior half field for the lower cervical and supraclavicular region were also included. All patients adopted simultaneous modulated accelerated radiation therapy (SMART) technology and external radiotherapy was given 2–2.47 Gy per fraction (Gy/F), once daily, five fractions per week (F/W). A total dose (TD) of nasopharygeal region was 64–74 Gy, the TD of metastasis lymph nodes was 60–68 Gy, and 56–66 Gy for CTV1, 54–58 Gy for CTV2, all of above divided into 28–33 fractions. All patients have no additional boosts and were treated with radical intent.

All 1912 patients that treated at Sun Yat-sen University Cancer Center (SYSUCC) between October 2009 and December 2010 were assessed using these criteria. In total, 625 patients received IMRT-based comprehensive treatment, of which 321 fulfilled all of the criteria above and were included in this retrospective analysis.

The protocol of this study was approved by the institutional review board of SYSUCC, and informed consent was obtained for each participant.

### End-points and statistical methods

Radiation treatment time (RTT) was defined as the duration from the initiation of radiotherapy to the day when the prescribed courses were completed. Duration of interruption (ΔRTT) was calculated as the number of days beyond the scheduled treatment time for the prescribed radiation course.

The primary endpoints of this study were loco-regional failure-free survival (LRFS), distant failure-free (DFFS) and progression free survival (PFS). Time to events in this study was determined from initiation of treatment to the event of interest or the end of follow-up (June 1, 2015). Persistent disease that documented 3 months following treatment and recurrence which happened in the nasopharyngeal region and/or neck lymph node were considered loco-regional failure. Patients were censored if no events occurred by last follow-up.

For this analysis, receiver operating characteristic (ROC) curves analysis cannot provide a meaningful cutoff, as it does not account for the time at which events occur. Therefore RTT and ΔRTT were analyzed as dichotomous variables in univariate analysis, using the lower quartile (P25), the median (P50) and upper quartile (P75) values as cut-off points. And in multivariate analysis they were performed as a continuous variable; this analysis was performed in similar fashion to the work of Sher et al. [32] and Cannon et al. [33].

Independent prognostic factors were identified using Cox's proportional hazard regression model. The distributions of patient characteristics among groups were assessed using the *t*-test for continuous variables and Chi-square test or Fisher’s exact test for categorical variables. All analyses used the conventional P < 0.05 level of significance.

### Follow-up

Outpatient check-ups were the main approach of follow-up. All patients received at least 3 months of follow-up after completion of treatment; because the duration is necessary for adequate assessment of the response after radiotherapy. Patients who returned to the clinic received a series of examinations: blood biochemical analysis, nasopharyngeal fiberoptic endoscopy, chest X-ray, abdominal sonography and MRI. Bone scans and PET-CT were performed for patients with suspected metastases. If patients did not return to the clinic, follow-up information from the patients themselves, their families, or the household registration office was obtained mainly by phone.

## Results

The clinical characteristics and treatment information for the 321 patients who met the study criteria are summarized in Table 1. Median age was 45 years (range, 11–77 years). Median follow-up was 58.3 months (range, 5.2–67.8 months) and 94.7% of patients were followed-up for more than 3 years. The male-to-female ratio was 3:1. The median total dose to the nasopharynx (NP) was 68 Gy (range, 64–74 Gy), and the median fractionated dose was 2.27 Gy/F (range 2.0–2.47 Gy/F). 68 Gy over 30 F for NP is the most commonly-prescribed dose (241, 75.1% of patients) in the present study. All patients completed the prescribed radiation course. Of the 321 patients, 228 (71.0%) also received platinum-based concurrent chemotherapy and 93 (29.0%) patients underwent radiotherapy alone.

**Table 1:** Clinicopathological features of the 321 patients with NPC.

Characteristic	No.	N%
**Sex**		
**Female**	**80**	**24.9%**
**Male**	**241**	**75.1%**
**7th AJCC Stage**		
**I**	**24**	**7.5%**
**II**	**70**	**21.8%**
**III**	**183**	**57.0%**
**IV**	**44**	**13.7%**
**T classification**		
**1**	**84**	**26.2%**
**2**	**63**	**19.6%**
**3**	**133**	**41.4%**
**4**	**41**	**12.8%**
**N classification**		
**0**	**54**	**16.8%**
**1**	**144**	**44.9%**
**2**	**119**	**37.1%**
**3**	**4**	**1.2%**
**CCRT**		
**Yes**	**228**	**71.0%**
**No**	**93**	**29.0%**
**Age (years)**	**Median (Range)**	**45 (11–77)**
**Follow-up (years)**	**Median (Range)**	**58.3 (5.2~67.8)**

Abbreviations: AJCC, American Joint Committee on Cancer; CCRT, concurrent chemoradiotherapy

**Fig 1:**
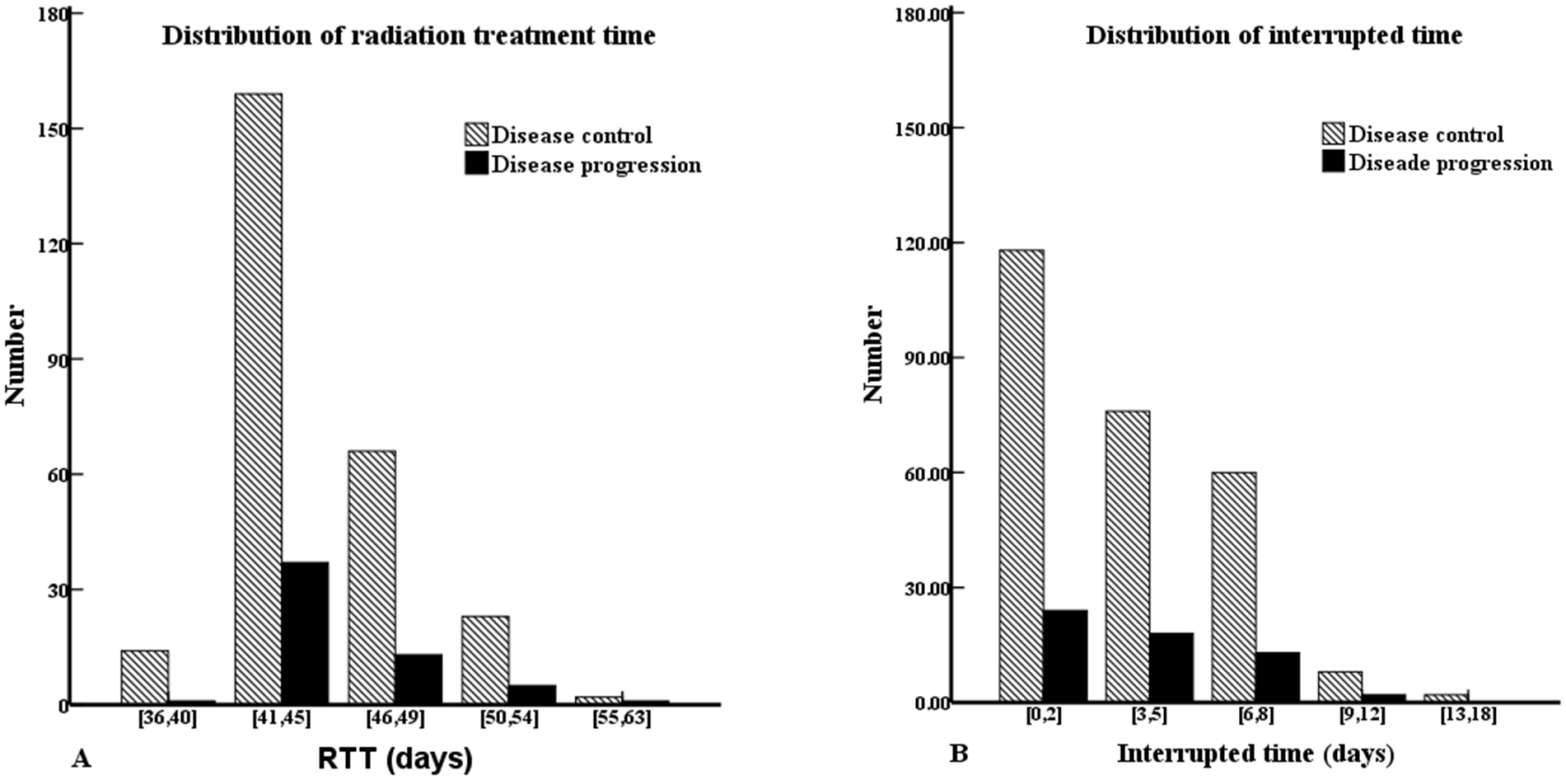
Histogram of (A) radiation treatment time and (B) interrupted time according to whether or not patients experienced disease failure.

For patients in whom the radiotherapy treatment time was prolonged, the ΔRTT was calculated as the actual time taken to complete the prescribed course of radiotherapy minus the scheduled treatment time. For example, assuming a prescribed dose of 68 Gy over 30 fractions, the scheduled treatment time was 40 days (starting treatment on a Monday) or 42 days (starting treatment on any day except Monday). The ΔRTT ranged from 1 to 18 days (median, 3 days). Nineteen of the 321 patients (6%) completed their prescribed course of radiotherapy ahead of the scheduled time (1–4 days) due to radiotherapists working overtime on holidays, only 31 (9.7%) patients finished their prescribed course of radiotherapy on time, and 271 (84.4%) patients had a prolonged RTT (1–18 days). The median RTT was 44 days (range, 36–63 days); 290/321 (90.3%) patients completed their course of radiotherapy within 7 weeks. The distributions of the RTT and ΔRTT values stratified by disease control are shown in Fig 1A and 1B.

The OS, PFS, LRFS and DFFS rates for the entire cohort were 90.7%, 82.1%, 88.9% and 89.4% respectively. A total of 57 (17.8%) patients developed disease progression, of which twenty four suffered loco-regional relapse, twenty three suffered distant metastases without loco-regional relapse, and ten suffered both loco-regional relapse and distant metastases.

### Progression-free survival

Five-year PFS for the entire cohort was 82.1% (95% confidence interval [CI], 77.9% to 86.3%). Univariate analysis of the associations between various clinicopathological factors and disease progression are summarized in Table 2. To analyze the impact of RTT and ΔRTT, patients were dichotomized using various cut-offs of those variables. P25 (43 days), P50 (44 days) and P75 (47 days) values are used in this study. According to the corresponding cut-off values, all patients in the study were divided into prolonged arm and relative on schedule arm. For example, to dichotomize the time parameter at RTT-P50 (44 days), two groups were created for comparison (RTT longer than 44 days vs. 44 days or less).

**Table 2:** Univariate analysis of factors associated with tumor control.

	PFS		LFFS		DFFS	
Variable	Rates	*P*	Rates	*P*	Rates	*P*
**T1-2 vs. T3-4**	**90.8% vs. 74.6%**	**0.000**	**92.7% vs. 85.5%**	**0.028**	**96.0% vs. 83.6%**	**0.000**
**N0-1 vs. N2-3**	**86.7% vs. 74.8%**	**0.005**	**92.1% vs. 83.8%**	**0.021**	**92.9% vs. 84.0%**	**0.017**
**I-II vs. III-IV**	**96.6% vs. 76.1%**	**0.000**	**96.6% vs. 85.6%**	**0.004**	**100% vs. 85.1%**	**0.000**
**CCRT (yes vs. no)**	**81.1% vs. 84.1%**	**0.418**	**88.7% vs. 89.3%**	**0.708**	**88.8% vs. 90.8%**	**0.550**
**RTT (≤ 43 d vs. > 43 d)**	**84.4% vs. 80.4%**	**0.417**	**88.9% vs. 88.8%**	**0.940**	**91.4% vs. 87.9%**	**0.395**
**RTT (≤ 44 d vs. > 44 d)**	**82.4% vs. 81.6%**	**0.943**	**88.6% vs. 89.1%**	**0.774**	**89.4% vs. 89.3%**	**0.890**
**RTT (≤ 47 d vs. > 47 d)**	**82.3% vs. 81.4%**	**0.839**	**88.4% vs. 90.9**	**0.588**	**89.3% vs. 90.0%**	**0.963**
**ΔRTT (≤ 1 d vs. > 1 d)**	**84.7% vs. 80.7%**	**0.375**	**89.3% vs. 88.6%**	**0.851**	**91.4% vs. 88.3%**	**0.451**
**ΔRTT (≤ 3 d vs. > 3 d)**	**81.7% vs. 82.6%**	**0.865**	**87.3% vs. 91.2%**	**0.276**	**89.7% vs. 89.0%**	**0.879**
**ΔRTT (≤ 6 d vs. > 6 d)**	**81.6% vs. 84.3%**	**0.640**	**88.4% vs. 91.7%**	**0.481**	**88.9% vs. 92.2%**	**0.528**

Abbreviations: CCRT, concurrent chemoradiotherapy; RTT, radiotherapy treatment time; ΔRTT, RTT minus time scheduled for the patient to complete the prescribed course of radiotherapy.

**Fig 2:**
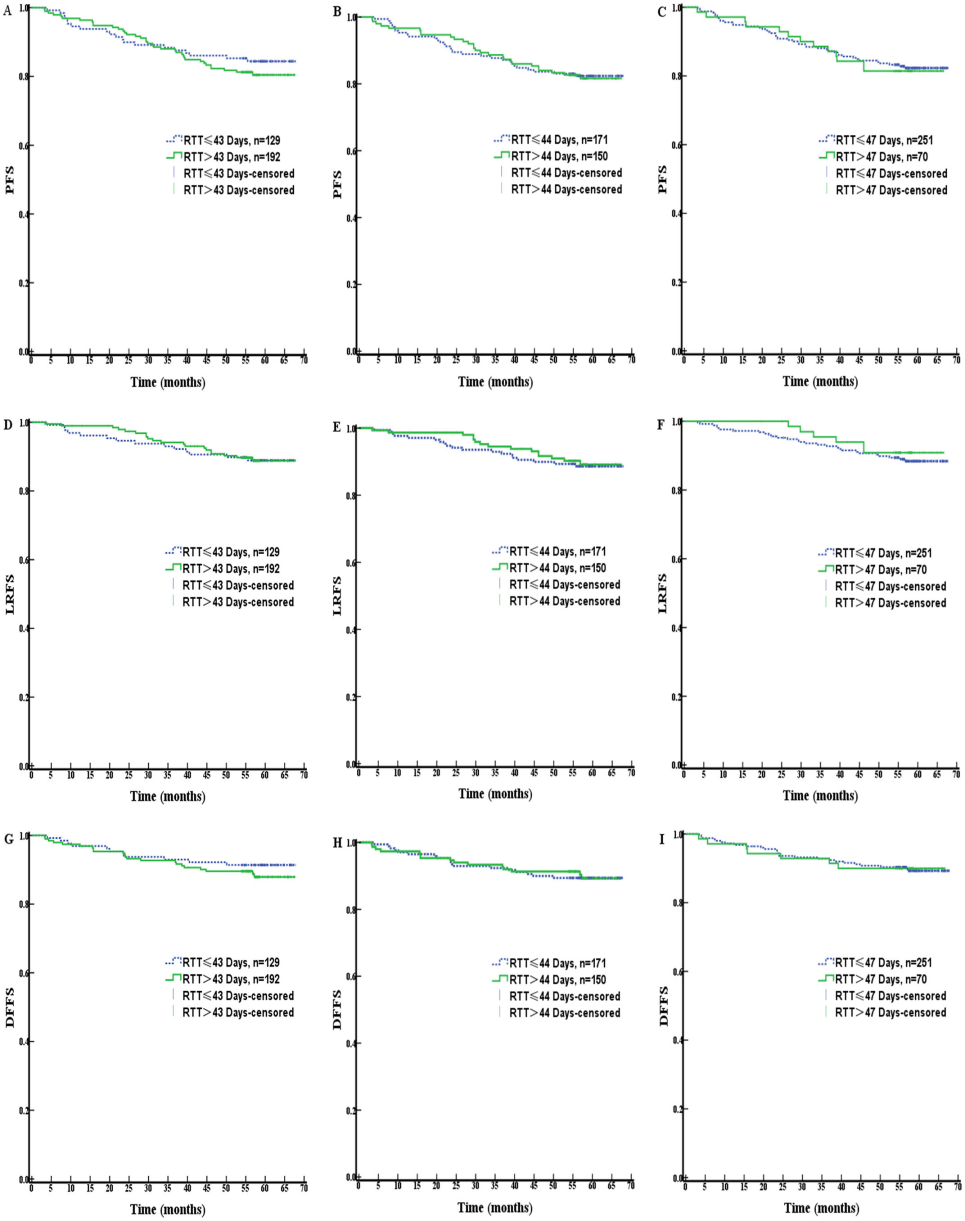
Progression free survival (PFS), Loco-regional failure-free survival (LRFS) and distant failure-free (DFFS) as function of number of RTT for NPC patients. (A-C): RTT ≤ 43 days vs. RTT > 43 days; (D-F): RTT ≤ 44 days vs. RTT > 44 days; (G-I): RTT ≤ 47 days vs. RTT > 47 days. (All P values > 0.05).

The difference between RTT-P25 (43 days) and RTT-P75 (47 days) was only 4 days, and no significant difference in PFS was observed for any RTT cut-off value (all *P* > 0.05). The PFS curves are shown in Fig 2A–2C. Patients’ characteristics and treatment modes are well-balanced in comparison arms when dichotomized by RTT = 43 and 44. And for RTT = 47, there was a larger proportion of patients staged T3-4 (46/70, 65.7%) in the RTT > 47 days group than the RTT ≤ 47 days group (127/251, 50.6%; *P* = 0.025). However, even after adjusting for T classification, no dramatic difference in PFS was observed between patients with a RTT > 47 days and those with a RTT ≤ 47 days (*P* > 0.05). With respect to ΔRTT, PFS was not significantly different when the patients were stratified using either the lower quartile (1 day), median (3 days) or higher quartile (6 days) as cut-off values (all *P* > 0.05). All comparison groups using the different cut-offs had an even balance of clinicopathological characteristics.

As excepted, larger tumors (T3-4) and more extensive nodal disease (N2-3) were associated with unfavorable PFS (T1-2 vs. T3-4: 90.8% vs. 74.6%, *P* < 0.05 and N0-1 vs. N2-3: 86.7% vs. 74.8%, *P* = 0.005). Concurrent chemotherapy was not significantly associated with PFS (*P* = 0.418). Patients who received concurrent chemotherapy in addition to radiotherapy had a similar average RTT compared to those who did not receive concurrent chemotherapy (44.8 vs. 45.1 days; *P* = 0.502). However, a significantly higher percentage of patients in the concurrent chemotherapy group had III-IVB disease (188/228, 82.4%) compared to the radiotherapy alone group (39/93, 42.0%; *P* < 0.05).

RTT was modeled as a continuous variable in multivariate analysis, and was not significantly associated with PFS (*P* = 0.408). Total radiation dose and the use of concurrent chemotherapy also have no significantly influence on PFS (*P* = 0.644 and *P* = 0.399 respectively). However, multivariate analysis confirmed that age was an independent prognosis for PFS, each additional year associated with an increase in the hazard ratio [HR] for PFS of 3.3% (*P* = 0.008). In addition, advanced T and N classification were independent prognostic factors for PFS (HR, 3.195; *P* = 0.001 for T3-4 vs. T1-2 and HR, 2.172; *P* = 0.004 for N2-3 vs. N0-1). In the multivariate analysis, ΔRTT-length of interruption-have not been entered into the Cox's proportional hazard regression model, due to the high correlation with RTT (correlation coefficient = 0.904, P < 0.001). The results of multivariate analysis are summarized in Table 3.

**Table 3:** Multivariate analysis of factors associated with tumor control.

	Hazard ratio (95% CI)	*P-*value
**PFS**		
**Age (per year)**	**1.033 (1.009–1.057)**	**0.008**
**T3-4 vs. T1-2**	**3.195 (1.642–6.220)**	**0.001**
**N2-3 vs. N0-1**	**2.172 (1.273–3.705)**	**0.004**
**RTT (per day)**	**1.033 (0.956–1.117)**	**0.408**
**Total dose (per Gy)**	**1.055 (0.840–1.325)**	**0.644**
**CCRT (yes vs. no)**	**0.750 (0.384–1.464)**	**0.399**
**LFFS**		
**Age (per year)**	**1.027 (0.996–1.059)**	**0.086**
**T3-4 vs. T1-2**	**2.169 (0.977–4.816)**	**0.057**
**N2-3 vs. N0-1**	**2.377 (1.182–4.780)**	**0.015**
**RTT (per day)**	**1.000 (0.903–1.108)**	**0.995**
**Total dose (per Gy)**	**1.238 (0.973–1.575)**	**0.083**
**CCRT (yes vs. no)**	**0.750 (0.321–1.751)**	**0.506**
**DFFS**		
**Age (per year)**	**1.032 (1.001–1.064)**	**0.043**
**T3-4 vs. T1-2**	**5.603 (2.032–15.450)**	**0.001**
**N2-3 vs. N0-1**	**2.205 (1.092–4.451)**	**0.027**
**RTT (per day)**	**1.017 (0.914–1.132)**	**0.758**
**Total dose (per Gy)**	**0.801 (0.541–1.184)**	**0.266**
**CCRT (yes vs. no)**	**0.629 (0.259–1.529)**	**0.306**

Abbreviations: HR, hazard ratio; CI, confidence interval; CCRT, concurrent chemoradiotherapy; RTT, radiotherapy treatment time; ΔRTT, RTT minus time scheduled for the patient to complete the prescribed course of radiotherapy.

### Loco-regional failure-free survival

A total of 34 patients (10.6%) experienced loco-regional recurrence, with a 5-year local control rate of 88.9% for the entire cohort (95% CI, 85.5% to 92.3%). The results of univariate analysis assessing the risk factors associated with LRFS are shown in Table 2. A significantly increased risk of loco-regional recurrence was observed for patients with advanced T and N classifications (T1-2 vs. T3-4; 92.7% vs. 85.5%, *P* = 0.028 and N0-1 vs. N2-3: 92.1% vs. 83.8%, *P* = 0.021). Similarly to PFS, all cut-off values for RTT and ΔRTT as dichotomous variables and chemotherapy were not significantly related to LRFS (all *P* > 0.05). The LRFS survival curves for all comparison arms distributed by various RTT cut-offs are shown in Fig 2D–2F. In multivariate analysis of factors potentially affecting loco-regional control, RTT was modeled as a continuous variable and was not significantly associated with LRFS (*P* = 0.995). Age, total dose and usage of concurrent chemotherapy were not significantly associated with loco-regional control (*P* = 0.086, 0.083, 0.506, respectively). However, N classification was an independent prognostic factor for LRFS (HR, 2.377; *P* = 0.015 for N2-3 vs. N0-1).

### Distant failure-free survival

A total of 33/321 patients (10.3%) developed distant metastasis, with a 5-year DFFS rate of 89.4% (95% CI, 86.0% to 92.8%) for the entire cohort. In univariate analysis, patients with early T and N classifications (T1-2, N0-1) had significantly better DFFS in comparison with patients staged T3-4 or N2-3 (*P* < 0.05 and *P =* 0.017 respectively). Univariate analysis of RTT and ΔRTT as dichotomous variable revealed no significant associations between prolonged treatment and DFFS (*P* > 0.05 for all). The DFFS curves as function of RTT are shown in Fig 2G–2I. Concurrent chemotherapy was not significantly associated with DFFS (*P =* 0.550).

In multivariate analysis, RTT had no significant association with DFFS (*P =* 0.758) as well. Increasing total dose and concurrent chemotherapy also gain no significant benefit in DFFS (*P =* 0.266 and 0.306 respectively). Similarly to the analysis for PFS, age, T classification and N classification were identified as independent prognostic factors for DFFS. These results are summarized in Table 3. Patients with a T3-4 classification had a HR of 5.603 for distant metastasis compared to those with a T1-2 classification (*P =* 0.001), and N2-3 stage also predicts poorer DFFS with a 2.205-fold greater risk of disease metastasis than N0-1 stage (*P =* 0.027).

## Discussion

Whether a prolonged RTT can affect survival outcomes in patients with NPC has not been extensively studied in the IMRT era. Prolonged RTT (ranging between 36 and 63 days) had no detrimental effect on PFS, LRFS or DFFS both in univariate and multivariate analysis in this retrospective study, and ΔRTT—as a surrogate for the duration of treatment interruptions—had no significant association with PFS, LRFS or DFFS in both analyses as well.

A review of the past literatures showed conflicting conclusions. Kwong et al. (27) from Hong Kong retrospectively assessed 796 patients treated with 2DRT, of whom 229 (28.8%) underwent a continuous course of radiotherapy (3.5 Gy/F, 3F/W, NP: 59.5 Gy/17F, Neck: 52.5 Gy/15F, 5-weeks for early-stage disease) and 567 (71.2%) patients received a split course (phase I: 2.5 Gy/F, 4F/W, NP and upper neck: 40 Gy/16F, 4week; 1 week as planned gap was allowed; phase II: 3.5 Gy/F, 3F/W, NP: 21 Gy/6F, Neck: 14 Gy/4F; 2 weeks for advanced-stage disease). No significant influence of radiation therapy time on outcome was observed in the continuous course subgroup. However, patients with a prolonged overall treatment time (delayed for one week or more) had significantly poorer loco-regional control and disease-free survival in the split course subgroup. Multivariate analysis showed each extra day increased the HR by 3.3% for local control and 2.9% for disease-free survival. The overall treatment time in the study did not include the time required for additional boosts, ranged from 38 to 80 days for the spilt course group and 37 to 82 days for the continuous course group. A study from Taiwan had showed similar relationship between the length of treatment and tumor-related outcomes (External radiotherapy:1.8 Gy/F, 5 F/W, 46.6 Gy/26F, 6 MV photon beam; Boost: 16.2–25.3 Gy/9-14F, 10MV photon beam; Intracavitary brachytherapy: 5–16.5 Gy/1-3F) [34]. A radiation time of no more than 12 weeks and radiation dose not exceeding 75 Gy were recommended to provide the optimal local control and disease free survival. However, the radiotherapy treatment time (median, 11.6 weeks, range, 7.8–20 weeks) including intracavitary brachytherapy time was considerably longer compared to other studies [27–31, 35]. In addition, several studies (fractionated scheme: 1.7–2 Gy/F, 5 F/W, 60–84 Gy/30-47 F) from China illustrated that prolonged treatment time and interruptions result in poor local control and survival rates in conventional 2DRT [28–31]. As is aforementioned, both in split course of hypofractionation and conventional fractionation radiation therapy, elapsed time during treatment reduced the local control rates and/or overall survival.

Gratifyingly, the introduction of IMRT for the treatment of NPC seems to have negated the influence of a prolonged RTT on treatment outcomes. Su SF and his colleagues retrospectively, analyzed 850 patients with NPC treated with IMRT (GTV: 68 Gy, CTV1: 60 Gy, CTV2: 54 Gy, in 30 F) and found no significant detriment in local control despite a prolonged RTT—within the range of 39 to 67 days overall—whether in radiation alone group or CCRT group [36]. The results of the present study concur with this analysis.

Whether prolongation would influence the therapeutic effects of radiotherapy in NPC, there is a big difference between 2DRT age and IMRT era. The following factors may explain the disparity. In the first place, $EQD_{\begin{array}{l} 2 \end{array}}=Total\;dose\times \frac{\alpha /\beta +Fractionated\;dose}{\alpha /\beta +2}$, *EQD*_2_ means equivalent total dose at 2 Gy/ F in conventional fractionation of altered fractionated condition. Using the commonly accepted assumption of an α/β ratio equal to 10, the calculated *EQD*_2_ of IMRT protocol were 64.00 Gy-76.88 Gy (median 69.51 Gy), almost equal to 70 Gy. In all studies about 2DRT mentioned above, the planed overall radiation therapy time is about 7 weeks, or even longer. While simultaneous modulated accelerated radiation therapy technology is used in IMRT age, the prescribed course in this cohort ranging from 29 to 33 fractions can be completed in about 6 weeks. The application of new segmentation model enables to shorten the total treatment time without decreasing total dose, so will cut down the time of tumor accelerated proliferation and reduce the number of proliferated tumor cells. On the other hand, the fractionated dose of IMRT (median 2.27 Gy) is larger than those of conventional fractionation (1.7–2 Gy), which is conductive to suppress reparation of sub-lethal damage. Reports about laryngeal carcinoma also have demonstrated that superior local control rates can be found when using 2.1–2.25 Gy/F along with a shorter treatment time compare to those with 2 Gy/F [17, 23, 37, 38]. In IMRT era, the reduced radiation therapy time render higher chance of breaks to patients when facing emergencies or serious treatment toxicity. Secondly, platinum-based chemotherapy not only improves loco-regional control by directly killing tumor cells and enhancing radiosensitivity, but also improves PFS by controlling distant subclinical metastatic foci. Thereby the application of concurrent chemotherapy may counteract accelerated repopulation to a certain extent. Thirdly, sub-lethal damage is the dominant effect on tissue of X-rays, for it is the low liner energy transfer (LET) ray. Theoretically, intervals will permit the repair of sub-lethal damage in normal cells. For patients with heavy acute reactions, temporary treatment gap may remit the symptoms and improve the tolerance of treatment. Furthermore, the casual breaks may grant time for tumor fading, which will promote the reoxygenation of tumor cell and then increase the radiosensitivity of tumor cells. Hence, the harmful effects of prolongation of RTT (repopulation) may be compensated for by these reactions. However, longer interruption will result in lower the biological effective dose according to the correction formula of $BED-cBED=EQD_{2}-\left(0.693/\alpha \right)\times \frac{T-T_{k}}{T_{pot}}$ where *T*_*k*_ is 28 days (the assumed lag period for a burst of accelerated repopulation of tumor clonogenic cells to occur), *T* is radiation therapy time, and *T*_*pot*_ is potential doubling time. Therefore, it is important to keep a balance between tumor control and RTT. Indefinite extension of RTT is not a wise option.

With regard to the effect of CCRT, several prospective randomized trials have illustrated that it is superior to radiation alone for the management of stage II–IVB NPC [39–43]. Therefore, it is worth noting that concurrent chemotherapy had no significant impact on PFS, LRFS or DFFS in this study. This may be attributable to the fact that the subgroup who received concurrent chemotherapy in this study contained a significantly higher proportion of patients with advanced stage (III-IVB) disease.

Several aspects must be considered to assess the reliability of this study. Firstly, the results of the univariate and multivariate analysis of the three tumor-related outcomes (PFS, LRFS and DFFS) remained consistent and provide confidence in the results. Secondly, confounding effects were controlled for, by limiting the included population and performing multivariate analyses. The present research excluded patients who received induction chemotherapy to eliminate interference, considering there may be phenomena of residual radiation resistant tumor cell, the increasing proportion of hypoxia cells after induction chemotherapy and delayed radiotherapy led by serious complication—although not been confirmed by relevant research. All patients in the present series were assessed using MRI, and also received chest X-rays, abdominal sonography, a bone scan and, in some cases, even PET-CT to confirm M0 disease.

However, given the inherent limitations of retrospective analysis, caution needs to be taken when interpreting these results. The cut-off of radiation therapy time is 50 days in the research of Wu et al.[30] and 56 days in Tan’s [31]. In the present study, 290 (90.3%) patients completed their course of radiotherapy within 7 weeks. The vast majority of patients who had "prolongation" completed treatment within several days after the scheduled date. The range of total days elapsed in their study went up to 63 days, but only a small minority of patients had prolonged treatment to this extent. Therefore, the present study may not be able to find the influence of prolongation. Furthermore, the present study did not identify an exactly safe RTT range, which merits further studies to explore more reliable limits for treatment interruptions. Another possible explanation for the lack of significant associations between the survival outcomes and prolonged RTT in this study may be the limited statistical power associated with the relatively small cohorts of patients and low numbers of treatment failure events.

## Conclusion

We conclude that no such association between survival outcomes and radiation treatment duration (range: 36–63 days) can be found in the present retrospective study, however, we have to remind that prolongation in treatment should be limited in clinical application and interruptions caused by any reason should be minimized as much as possible. Because the article does not find an exactly safe and reliable reference range, further multiple institution study and longer follow-up of larger populations or even prospective randomized study is urgently needed to confirm the results and seek to a safe range.
